# Pan-genomics: Insight into the Functional Genome, Applications, Advancements, and Challenges

**DOI:** 10.2174/0113892029311541240627111506

**Published:** 2024-07-03

**Authors:** Akansha Sarawad, Spoorti Hosagoudar, Prachi Parvatikar

**Affiliations:** 1 Department of Biotechnology, Applied School of Science and Technology, BLDE (DU), Vijayapura, Karnataka, India

**Keywords:** Pan-genomics, telomer genome, human genomics, evolution, OMIM, conventional genomic

## Abstract

A pan-genome is a compilation of the common and unique genomes found in a given species. It incorporates the genetic information from all of the genomes sampled, producing a big and diverse set of genetic material. Pan-genomic analysis has various advantages over typical genomics research. It creates a vast and varied spectrum of genetic material by combining the genetic data from all the sampled genomes. Comparing pan-genomics analysis to conventional genomic research, there are a number of benefits. Although the most recent era of pan-genomic studies has used cutting-edge sequencing technology to shed fresh light on biological variety and improvement, the potential uses of pan-genomics in improvement have not yet been fully realized. Pan-genome research in various organisms has demonstrated that missing genetic components and the detection of significant Structural Variants (SVs) can be investigated using pan-genomic methods. Many individual-specific sequences have been linked to biological adaptability, phenotypic, and key economic attributes. This study aims to focus on how pangenome analysis uncovers genetic differences in various organisms, including human, and their effects on phenotypes, as well as how this might help us comprehend the diversity of species. The review also concentrated on potential problems and the prospects for future pangenome research.

## 1 INTRODUCTION

In the early stages of the genomic era, individual genomic references serve as the foundation for understanding variations in inheritance among organisms [1]. Examining sequencing reads aligned with a reference genome can provide valuable insights into scrap variants such as Single Nucleotide Polymorphisms (SNPs) and small Insertions and Deletions (indels), leading to a better understanding of natural heritable diversity, population history, and genome-based ancestry [2-4].

Genomic variation includes SNPs, indels, and other structural variants like CNVs, rearrangements, and P/A variants. Even though structural variants make up a small percentage of genomic variation, they can affect between 4 and 12 percent of disease-related genes by altering gene function and disrupting gene function in humans (10-12%). Using a single reference genome can lead to missing a significant amount of inheritable information due to the inheritable variability across members of the same species [5].

Pan-genome technology can be employed to determine numerous further generous variation signals during the isolation of species, and it's a foremost approach for genomic exploration and parentage [6]. Gratuitous genes, in particular, are constantly tightly linked to the development and environmental rigidity of significant agronomic features [7]. The pan-genome sequencing of germplasm that contains distinguishing traits similar to those seen in wild and domesticated species may help reveal the lost inheritable diversity during domestication and provide information on parentage. An increase in the number of species having their genomes sequenced has been brought on by the development of high throughput sequencing technologies [8].

The original pangenomes were performed in bacteria, and the changeable genomic factors were associated with bacterial aridity genes and some non-imperative natural pathways, which were helpful for subtyping various bacteria and creating vaccines [9, 10].

As of December 2014, more than 40 bacterial species have more than 20 fully assembled genomes from diverse strains and isolates, allowing for extensive pan-genomics analyses [11]. The complete genetic data for a species or group is modeled by a pangenome. In contrast to reference-based methods, which associate sequences with a particular reference genome, pan-genome reference systems strive to define the interaction between all represented genomes [12].

## 2 CONCEPT OF PANGENOME

The term “pangenome” is derived from the molecular analysis of prokaryotes and basically describes the entire set of transcribed genetic units in each branch of a monophyletic (bacterial) group that is currently known [13-15].

Assembling all the genomes of a phylogenetic clade would yield the outcome of making all the genes for that group in a higher genetic structure that could circumscribe its entire genetic variety and storehouse in a conceptual object dubbed a “super genome” [16]. As a result, sequencing every genome from that species is the only way to ascertain its complete genetic makeup. When it comes to a collection of specimens that, in theory, share an ancestor, the pan-genome is the comprehensive list of all genetic changes for that specimen [17]. Even though the bacterial genome is small, haploid, and almost devoid of introns or intergenic DNA, its transcriptional unit composition is remarkably flexible because only a small number of genes are absolutely necessary for the bacterium to function, while a larger group of accessory genes may mediate survival in particular environmental conditions and other collateral properties (for example, antimicrobial resistance or toxin production) [18-21]. As a result, the persistence of this adaptable genomic material, which is unique to prokaryotes and makes up the “pan-genome,” is what ensures rapid evolution and adaptability as well as great plasticity [20]. On the other hand, multicellular Eukaryotic organisms frequently have numerous copies of the same DNA strand and DNA fragments in each of their cellular nuclei [21].

**Fig. (1):**
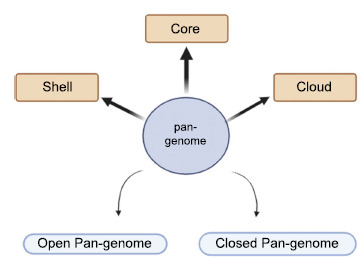
Organization of a pangenome composed of core and dispensable components of the genome.

In human genetic sequence, virtually every gene is present, but not all of them are actively working, and these many copies carry comparable coding information. The complexity of Eukaryotic DNA, on the other hand, is found in complex multicellular animals and is significantly concentrated in non-genic material. It also demonstrates a higher level of structural diversity, which exceeds individual variances in single nucleotide polymorphisms [22-25]. Because the presence or absence of a gene cannot serve as a useful descriptor of genomic variability in high-order groups of Eukaryotes, a more inclusive representation should acknowledge that genomic variability includes point mutations in addition to insertions and deletions, repetitive DNA, mobile genetic elements, inversions, duplications, and gene fusions (Fig. **1**) [26].

### 2.1 Pan Genome in Bacteria

Bacterial genomes are influenced by population dynamics, which makes it important to estimate the effective population size accurately. However, determining this size for microbial species is challenging due to various factors such as the large size of their census populations, variations in recombination among lineages, and the criteria for defining a species [27].

Bacteria have the potential for significant population growth due to their small size, asexual reproduction, and rapid generation rates. These factors allow them to reach high population densities even in restrictive environments. It is important to note that in most bacterial species, there may be significant differences between the standing population and the effective population size, as the former is often established by one or only a few individuals [28].

An enormous variety of bacterial genomes have been produced since the development of sequencing technology. Theoretically, a species can be described using one or more of these genomes; however, it is still unclear how many genomes were required to characterize a species of bacteria properly. Tettelin *et al.* investigated this question in 2005 by comparing the genomes of eight distinct bacterial strains [29]. They were the first to suggest using the pan-genome idea to identify a particular bacterial species. A core genome and a nonessential genome were present in this pangenome. The majority of housekeeping genes, which are unknown in other genomes constructed later, were included in the core genome [30].

The pangenome of bacteria suggests that the available data for comparing genetic diversity across multiple individual genomes is restricted. Therefore, additional genetic data is needed to describe a species' rich diversity and biological characteristics. Nonessential genes, in contrast to those found in the core genome, are those that contribute to the diversity of bacterial species and engage in biochemical pathways and functions that are not necessary for bacterial growth. These activities frequently lead to selection advantages, such as the ability to adapt to various ecological environments, develop resistance to antibiotics, or colonize new hosts. This shows that pangenome analysis can be used to find a huge number of variable novel genes of value and research significance (Table **1**) [31, 32].

### 2.2 Pan Genome of *Escherichia coli*

*Escherichia coli*, the most well-known bacterium, has a close relationship with its human host and plays a significant role in industrial microbiology and biological engineering. While the exact connection between *E. coli* and humans is not fully understood, it has important implications for the prevention of illnesses and the maintenance of good health. Increasing the conversion efficiency of the target product can be achieved by utilizing the minimal gene set of a viable *E. coli* strain in industrial production. Over a long period of evolution, *E. coli* has developed a mutually beneficial symbiotic relationship with the human host, resulting in the steady generation of local optimal fitness, encompassing several features [33].

Accessory genes are required for adaptive evolution and the ability to respond to environmental changes. As a result, it is critical to undertake an association analysis of accessory genes to identify those that are specifically related to *E. coli*'s adaptation to its human host. These interactions not only influence the successful colonization of a human host by an *E. coli* population but also have a direct impact on the host's growth, physiology, and health [34].

Numerous experiments have been conducted to reduce the size of the *E. coli* genome. In 2006, Posfai *et al*. created the Multiple-Deletion Series (MDS) strains by removing genomic areas present in strain K12 but lacking from five other strains. Among these, the MDS42 strain has demonstrated significant application value and is now commercially available [35]. In 2011, Iwadate *et al*. developed the *E. coli* strain 33, which had a genome up to 38.9% smaller than that of MG1655. This was achieved by deleting non-essential gene-containing chromosomal regions. However, the resulting strain showed poor development [36]. Hirokawa *et al.* (2013) created the strain DGF-298, which had a 35.2% reduction in the W3110 genome, by first removing the non-backbone regions identified through genomic comparison of strains W3110 and O157 Sakai, as well as some previously deleted and unknown genes. It should be noted that earlier attempts to create reduced strains relied on important genes discovered through experimental studies and comparative genomics of a small number of *E. coli* strains but lacked comprehensive genomic data [37].

### 2.3 Pan-genome in Fungi

Snow Mold (SM), a severe disease affecting winter cereals and grasses, is caused by fungi and fungal-like organisms exhibiting phytopathogenic and psychrotolerant/psychrophilic characteristics. This disease is predominantly found in the Northern Hemisphere, particularly in regions such as Europe, Canada, the United States, the United Kingdom, and Japan. The sequencing of numerous phytopathogenic fungi genomes has led to significant progress in the research and management of these organisms [38]. For instance, whole-genome sequencing of *Ustilago maydis*, *Botrytis species*, *Zymoseptoria tritici*, and *Rhizoctonia solani* unveiled genes associated with effector proteins and mycotoxin-biosynthetic pathways. Whole-genome analyses have facilitated the creation of diagnostic markers to identify *Magnaporthe oryzae* and *Calonectria* species while also discovering markers that differentiate the aggressiveness levels of *Fusarium graminearum* strains for better diagnostic approaches [39]. Reference genomes are essential for transcriptome research focused on comprehending the physiological responses of fungi at a whole-genome scale. Utilizing the Funannotate program, Illumina mRNA-Seq experiment reads were mapped onto the assembled genome to pinpoint protein-coding genes in *M. nivale*. A total of 11,789 protein-coding genes were identified. An *in silico* Hidden Markov Model search predicted 138 tRNA- and 46 rRNA-encoding genes. An average *M. nivale* gene measured 2,033 base pairs in length. The assembly's long-range continuity was demonstrated by the L50 scaffold (N50 = 3,479,159 bp), which was approximately 1,711 times larger than an average gene size [40].

### 2.4 Pan Genome in Plants

The pan-genomes for major crops, like maize, rice, wheat, and soybean, based on high-quality genomes of multiple samples, have greatly advanced research into the evolution of plant genomes and the identification of critical genes linked to significant agronomic traits. De novo assembly of 54 inbred lines produced the model grass *Brachypodium distachyon *pan-genome, which was discovered to have almost twice as many genes as any single genome. The shell and softcore genes were enriched in functions that might be useful in particular contexts, while the core genes were enriched primarily in vital cellular functions. Consistent with findings for other plant pan-genomes, this study showed that transposable elements were important in the evolution of the genome and that gene PAVs contributed significantly to phenotypic diversity [40].

Utilizing a map-to-pan approach, the first cotton pan-genome was created from the re-sequencing data of 1961 cotton accessions. This variation repertoire showed that genomic divergence during cotton domestication and improvement had influenced the identification of advantageous gene alleles for better breeding practices. The most varied cotton variations to date were found in this pan-genome, which also offered fresh concepts for precisely enhancing significant cotton features and the genomic underpinnings of cotton domestication [41].

The strawberry (*Fragaria spp.)* is an excellent model system for both basic and applied research due to its complex ploidy variations, unique mating systems, and exceptional nutritional composition (3,4). 128 individuals from 10 diploid species were re-sequenced, and the pan-genome of five diploid Fragaria species was created. The genetic diversity, demographic history, and natural selection of strawberry species were then analyzed. The MYB10 gene was shown to have many separate single base alterations that were linked to fruit with a white tint [42].

The ability to identify SVs without reference bias has never been better, thanks to the availability of several reference genomes within a species. The vast and complicated genomes of many crop species make genome assembly prohibitively expensive. At the moment, the “map-to-genome” method with short reads is mostly used to construct pan-genomes with large numbers of samples. This allows for the development of pan-genomes at comparatively lower sequencing depth and expense. Unfortunately, because crop genomes are extremely repetitive, it has been difficult to identify SVs because short reads are unreliable and inefficient in these areas [43, 44].

Bread wheat (*Triticum aestivum* L.) is among the most widely grown crops worldwide, yet meeting the escalating demands of an expanding global population poses significant challenges. With climate change anticipated to result in yield losses between 17-31% by the mid-21st century, it becomes imperative to develop improved genomics-based breeding strategies for creating climate-resistant wheat varieties [45]. Wheat genomics has seen rapid advancements in recent years, highlighted by the first draft genome assembly in 2014 by the International Wheat Genome Sequencing Consortium (IWGSC) utilizing shotgun sequencing of isolated chromosomal arms. In 2018, the IWGSC published a final reference genome assembly after producing the first nearly complete assembly of the “Chinese Spring” cultivar in 2017, along with assembling 15 additional cultivars from global breeding programs. The Chinese Spring reference cultivar displayed the most unique segments, encompassing 158,503 segments found solely in this variety. The IWGSC functional annotation identified these genes as highly variable ones, featuring transposable elements or transposable element candidates and disease resistance genes with an NB-ARC domain, compared to other cultivars. This data indicates that modern cultivars may have lost these genes in comparison to Chinese Spring varieties [46].

**Fig. (2):**
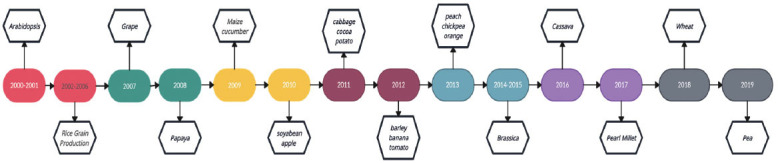
Timeline of pangenomics era of plant.

Plants have been subjected to genomic hybridization for more than a century to produce offspring with desirable characteristics. The incorporation of genes from wild plant varieties that enhance important properties of crops, such as appearance, nutrient content, resistance to pests or diseases, as well as tolerance to conditions like drought or heat, is now a common practice in the breeding of commercial crop varieties [47]. Large-scale genomics projects are underway to investigate plant genetic diversity, which includes not only the model plant Arabidopsis thaliana but also a variety of crops. For example, hundreds of thousands of kinds of rice, maize, sorghum, and tomato are being re-sequenced. In plant breeding, a pan-genome structure provides various advantages over a single, linear reference genome sequence [48]. By incorporating the genome sequences of wild relatives, a pan-genome provides a unified coordinate system for all known variations and phenotype information of a specific crop, enabling the identification of novel genes that are absent in the reference genome(s). Furthermore, the pan-genome allows for the detection of chromosomal rearrangements among different genotypes, which can impede the introduction of desired genes. Additionally, it offers a concise representation of polyploid genomes and facilitates the quantification of allele dosage in the case of autopolyploid (Fig. **2**) [49].

### 2.5 Pangenome of Animals

Relatively few studies have been dedicated to exploring the animal pangenome, which primarily focuses on the field of population genetics and mutation processes. In contrast to animals, plants have experienced numerous instances of polyploidization, resulting in a more diverse range of strains, complex agricultural characteristics, and larger effective population sizes. At present, the animal pangenome relies on extensive comparative genomics to detect variations in animal genomes and discover genes that are exclusively expressed, thereby shedding light on animal origins, evolution, and phenotypes [50].

The initial investigation on this topic, which was released in 2017, examined the genetic makeup of nine distinct pig breeds from different regions in Asia and Europe. This study revealed a significant amount of fresh genetic variations and 137.02 Mb of previously unidentified sequences. In comparison to the pangenome, Chinese pigs displayed a high occurrence of approximately 9 Mb of pan-sequences that encompassed the tazarotene-induced gene 3 (TIG3), a regulatory gene for adipose lipolase that is specifically expressed in Chinese pig breeds and promotes fat accumulation [51]. Additionally, a noticeable number of single nucleotide polymorphisms (SNPs) were observed in these shared sequences, and their composition varied significantly between males and females. The construction of the pig pangenome provides insight into the genetic diversity that goes beyond the information provided by the reference genome. Pangenomic research on genetic variation can simplify the identification of previously undetected mutations and expedite subsequent genome analyses [52].

Apart from their genetic composition, the transcriptomes, proteomes, and metabolomes of human cells exhibit diversity. Consequently, a single human tissue sample may contain various genetic patterns. This genetic heterogeneity is evident in the array of cell populations present, such as the immune system's T cells that have been studied at the single-cell level. The detection of genes associated with specific cellular traits and the genetic disparities among different human cell types can be achieved through pan-genome analysis. Utilizing the pangenome as a powerful instrument aids in understanding the intricacies of human cells and developing new treatments for numerous human diseases [53].

The challenge of gathering the best possible samples has risen because of the unique characteristics of the location and the way that animals and fowl have been domesticated. As a result, domestic animal pangenome research has stagnated. Pigs were the first among them to be the focus of pan-genomics research. There were a lot of functional genes that are biologically meaningful among the 1.3%–14.9% of novel sequences discovered in the instances that are currently available. These genes are primarily enriched in relation to different species' immunological responses, suggesting that domestic animals can use these genes to increase their resilience and better adapt to harsh situations like cold and heat. Furthermore, through the validation of various WGS data, the pangenome reference model has improved its capacity to distinguish between SVs [54].

**Fig. (3):**
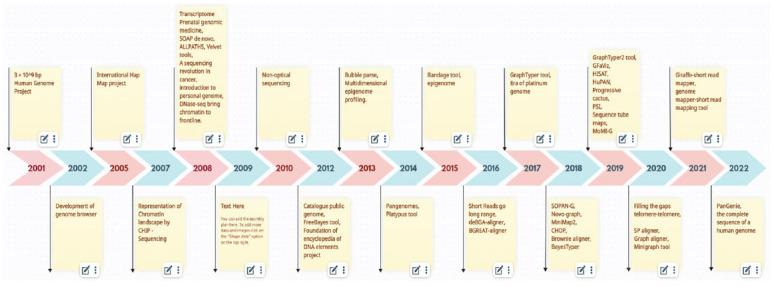
Timeline of pangenomics era.

Numerous SVs found in this reference model were linked to significant biological traits in poultry or cattle as well as to advancements in domestication [45]. The biological variants that will come to light will enable us to make the most use of these genetic resources and will aid in our deep understanding of the hidden mechanisms driving various phenotypes. The long-standing ban on utilizing SNPs and indels for hereditary analysis is broken by the SV sets and novel sequence variants created in light of the pangenome. This will provide a new method for analyzing the genetic makeup of cattle and poultry breeds worldwide (Fig. **3**) [55].

### 2.6 Pangenome of Human

Developing an in-depth knowledge of how genotype affects phenotype is a major goal in human genetics. For rare genetic illnesses, where a single mutation typically causes disease, several genes have been effectively identified to date. A few thousand of these disease genes are currently annotated in the Online Mendelian Inheritance in Man database. Linkage analysis in affected pedigrees has shown to be a highly successful method for localizing causal genes for mutations with completely penetrant effects [56]. Whole-exome sequencing in families has recently emerged as a complementary and potentially more successful tool for discovering the causative mutations in novel disease genes and, in certain cases, confirming the significance of de novo mutations in affected children. Such variants can be implicated as pathogenic based on their frequency in the population by comparing identified variants in affected family members to those found in the general population. Common diseases have traditionally been more difficult to study because they are caused by the combination of several genetic and nongenetic risk factors, each of which only slightly raises the overall risk. GWAS has discovered thousands of strong genotypephenotype relationships for a wide range of human traits and illnesses [57]. Catalogs of human genome variation, linkage disequilibrium characteristics, and the commercial development of low-cost microarrays have all contributed to this success. This emphasizes the significance of building pan-genomic resources to incorporate common and rare variants for imputation and association testing of regularly segregating variations, as well as the interpretation of uncommon variants in personal genome sequences. Despite efforts to reveal structural variation through discordant mapping of short reads, a considerable percentage is still ignored, largely because of their complexity, and the current reference genome is insufficient. It would be far preferable to genotype known SVs and reduce false positives among novel variants by including fully resolved, high-quality structural variation data into a pangenomic reference, especially from long-read sequencing data. This will be critical in the therapeutic setting, as genome sequencing is expected to replace array-based copy number variation studies over the next several years [58].

### 2.7 Pangenome Construction: Basic Approaches of Pangenome Construction

The sequencing unit for pangenome analysis can be anything from ORFs, genes, Clusters of Orthologous Groups (COGs), Coding Sequences (CDS), proteins, arbitrary sequence chunks, or protein entities, and so on. Pan-genome analyses are now possible thanks to advancements in “data structures,” in addition to cutting-edge sequencing technologies and experimental techniques. This section surveys current methods and identifies key design objectives for pan-genome data structures. Re-sequencing-based investigations have made use of this condensed representation of a set of transcripts for a particular gene, which helps to align RNA sequencing reads to the complete transcriptome [59]. Genomic sequences, observable connections between them and the original transcripts, and the reference genome that was utilized to construct the graph are all included. On the other hand, pan-genome data structure is used to transcribe sequences in order to best support the intended application of analyzing RNA sequencing data. On the one hand, this example shows that “computational pan-genomics” applies to collections of genetic sequences that are not necessarily whole genomes. However, this example emphasizes how crucial “graphs” are to pan-genomic data structures. The graph, in this case, is made up of sequences (nodes), the connections that connect them (edges), and the sequences that give rise to them (paths). The interaction of these fundamental components is necessary for a broad range of pan-genomic processes [60].

Construction includes the following steps: (1) currently available linear reference genomes and their variants; (2) haplotype reference panels; and (3) raw reads obtained either from multiple samples sequenced separately or from bulk sequencing of complex mixtures. The data structure should support local adjustments like the addition of a new genetic variant, the insertion of additional genomes, and the deletion of enclosed genomes, all without requiring a rebuilding of the complete data structure [61].

There are several methods for producing pangenomes, including “map-to-pan,” iterative assembly, and comparative de novo methods. and outlined the current methods used to create the plant genome. After identification, functional annotation is applied to the genes. With a few minor adjustments, this fundamental process serves as the foundation for the many pangenome construction strategies. While the other two approaches rely on creating a pangenome reference sequence, the comparative de novo approach uses the idea of comparing annotations of de novo genome assemblies of individuals to identify core and dispensable genes [62].

This method is best exemplified by early pangenome studies in rice and soybean. The pangenome sequences that were discovered are then annotated. Finally, the pangenome alignment of the mapping reads reveals the genic PAVs. In contrast, iterative assembly and map-to-pan approaches use different methodologies to generate a pangenome sequence. The former includes matching initial sample reads with the reference whole-genome assembly, whereas the latter updates the reference assembly by including unmapped reads. Iterative assembly and “map-to-pan” approaches benefit from low sequencing depth-based genic PAV identification by mapping short reads to an annotated genome; nevertheless, their applicability to simpler genomes with less repetitive gene sequences is limited [63].

Iterative assembly and “map-to-pan” techniques, which map short reads to an annotated genome, can find genic PAVs at low sequencing depths; however, their application is limited to smaller genomes with less repetitive gene sequences. To enable higher-quality annotation, the reference genome assembly must be big, complete, and appropriately fragmented. Long-read sequencing technology has the potential to alleviate the problem of fragmented assemblies caused by short-read sequencing technologies' incapacity to resolve repetitive sequences in complex genomes [53]. The fragmented assemblies result in low-quality functional annotation by underestimating the total number of genes and interfering with SV detection [64].

During pangenome analysis, gene mapping and assembly are critical considerations. Numerous strategies have been investigated. Several strategies can be employed to create a pan-reference for anchoring additional genes. One technique is to co-localize critical genes with disposable genes using synteny [65].

### 2.8 Different Tools and Databases Used in Pan-genome Analysis

In recent times, numerous captivating technologies have emerged to offer automated analysis processes. Each one targets a specific aspect of pangenomic functions for scrutiny. The primary analytical step is carried out by the Panakeia.py script. It reads the genome annotations of each input genome in GFF3 format and generates “strain graphs,” which consist of protein clusters as nodes connected to neighboring nodes through edges [66].

The Fastq reads were subjected to quality control using FastQC and filtered through Trim Galore (http:// www.bioinformatics.babraham.ac.uk/projects/trim_galore/). Genome assembly was performed using SPAdes. To enhance genome quality, only contigs longer than 500 bp were included in the assemblies. QUAST assessed the quality of these assemblies, while Prokka annotated the resultant scaffolds. The latter software package facilitates protein annotation by comparing sequence similarities with other proteins across various databases [67].

Roary v3.13.0 was used for pan-genome analysis, utilizing the GFF3 files generated by Prokka. The parameters included paralog division, a preset 100,000 clusters, and a 90% identity threshold for BLASTP [68]. Panaroo v1.3.3 in stringent mode was employed to determine the sizes of the core and accessory genomes. The query_pan_genome feature (-difference) aimed to identify exclusive accessory genes in either the EPTB or PTB symptoms. Finally, the gene_presence_absence.csv output files from Roary were analyzed by Scoary to associate the distribution of accessory genes with either the PTB or EPTB phenotype [69].

PanRV, the first all-encompassing automated process, has demonstrated its ability to accurately and effectively select potential vaccine candidates from species pangenomes. The pipeline is user-friendly due to its interactive graphical user interface and one-step installation method through the created installer [70]. A Linux-based Java package called PanRV was developed, specifically supported by the most recent version of Java and Ubuntu 14.04 and 16.04. However, PanRV has several dependencies and may require 15 GB of hard disk space. For efficient analysis of massive datasets, it is recommended to have at least 4GB RAM. PanRV provides an executable installer file (Installer.sh) at https://sourceforge.net/projects/panrv2/files/Installer.sh/download to assist with installation [71]. This feature is designed to cater to individuals with limited experience in calculations. Since this project is based on object-oriented programming, additional features may be added in the future to enhance functionality. GET_HOMOLOGUES, an open-source software program, harnesses well-known orthology-calling techniques, enabling non-bioinformaticians to obtain comprehensive and customizable pan-genome assessments of microbes [72].

By utilizing the HMMER3 program for scanning protein domain composition, adjusting desired alignment coverage cutoffs, or selecting syntenic genes only, the stringency of clustering can be adjusted. By calculating consensus clusters from the combination of clustering methods and filtering criteria, the resulting families of homologous genes can be further strengthened. Auxiliary scripts simplify the construction, analysis, and visualization of core genome and pan-genome sets. The generation of high-quality visuals can be achieved by fitting exponential and binomial mixture models to the data for determining theoretical core genome and pan-genome sizes [73].

A desktop-based software called Pangenome Analysis Toolkit (PATO) was developed for the simultaneous examination of thousands of genomes. In addition to its novel features for describing population structure, annotating pathogenic traits, and constructing gene sharedness networks, PATO also performs standard pan-genome analysis tasks such as defining the core genome and analyzing accessory genome attributes [74]. Designed in R, PATO integrates seamlessly with a wide array of tools available for genomic, phylogenetic, and statistical analyses. With remarkable speed, PATO completes even the most challenging bioinformatic studies in a matter of minutes without compromising accuracy. Furthermore, PATO encompasses all the necessary functions for comprehensive investigations of typical microbiology study objectives. It can be easily connected with other R analytical programs and offers tools for result visualization [75].

By utilizing the HMMER3 program for scanning protein domain composition, adjusting desired alignment coverage cutoffs, or selecting syntenic genes only, the stringency of clustering can be adjusted. By calculating consensus clusters from the combination of clustering methods and filtering criteria, the resulting families of homologous genes can be further strengthened. Auxiliary scripts simplify the construction, analysis, and visualization of core genome and pan-genome sets. The generation of high-quality visuals can be achieved by fitting exponential and binomial mixture models to the data for determining theoretical core genome and pan-genome sizes [76].

A desktop-based software called Pangenome Analysis Toolkit (PATO) was developed for the simultaneous examination of thousands of genomes. In addition to its novel features for describing population structure, annotating pathogenic traits, and constructing gene sharedness networks, PATO also performs standard pan-genome analysis tasks such as defining the core genome and analyzing accessory genome attributes [77]. Designed in R, PATO integrates seamlessly with a wide array of tools available for genomic, phylogenetic, and statistical analyses. With remarkable speed, PATO completes even the most challenging bioinformatic studies in a matter of minutes without compromising accuracy. Furthermore, PATO encompasses all the necessary functions for comprehensive investigations of typical microbiology study objectives. It can be easily connected with other R analytical programs and offers tools for result visualization [78].

The PanACoTA (https://github.com/gem-pasteur/PanACoTA) tool allows for downloading genomes from a species, creating a database with quality-checked and non-redundant genomes, uniformly annotating the pangenome, generating multiple variants of the core genomes, aligning the variants, and swiftly constructing a phylogenetic tree [79]. To compute the “pan-genome” based on comparisons at the genome and proteome levels, use PanGeT, the pan-genomics tool. With the use of graphical plots, this automated application effectively visualizes the entire pan-genome using LaTeX libraries. Links have also been given for retrieving sequencing data and functional annotations. Depending on the user's study objectives, this suite provides them the option to use either the BLASTN or BLASTP modes [80].

It will be advantageous for the users to have a variety of tools to choose from, based on the nature of their study requirements, despite the fact that there are other pan-genomic tools that are available, each with its own strengths and demerits. However, in order to assess the effectiveness of the suggested tool, PanGeT's core and strain-specific gene predictions were contrasted with those of PGAP, PanSeq, and GET_HOMOLOGUES (Table **2**) [81].

### 2.9 Limitation of Software/Tools

While a single tool may not always be able to meet the needs of comprehending the big picture, user wish lists are always beneficial in helping package developers prioritize their aims and give them guidance on how to improve each package.

The precision of genome assembly and annotation has a significant impact on pangenomic analysis performance. As a result, a sufficient quantity of finished sequence assemblies is required. Presently, the majority of bacterial genome sequences are, in fact, incomplete, and high-quality and high-coverage raw data are the only ones accessible for some of them. In order to incorporate partial genome assemblies for pan-genomic research, scaffold building and contig data file reformatting may be necessary. Even though third-generation sequencing technology is developing, which will undoubtedly aid in the assembly and completion of prokaryotic genomes, incomplete prokaryotic genomes are anticipated to be mass-deposited into public databases. If such data are not used, it would be a waste [82].

A crucial stage in pangenomic analysis is the identification of orthologous genes. The ortholog discovery tools available today primarily rely on sequence similarity, evolutionary relationships, or additional annotation data, including functional information. Pangenomic analysis accuracy can be significantly increased by developing a new and more effective ortholog identification approach for several closely related strains and isolates. Using gene gain-and-loss data to construct phylogenies between strains and isolates is one approach [83].

In a few ways, sampling is crucial for pan-genomics as well. One is the number of isolates or strains to select for a pangenomic study. For pan-genomic analysis, the other involves implementing a filter that separates more diverse strains or isolates from less diverse ones. The selection and reorganization of individual genomes are necessary to improve the average nucleotide identity (ANI) representation. One of the best metrics for separating species is ANI. Therefore, comprehensive data on the available samples—including their genotypes, phenotypes, and habitats—is crucial for a better pangenomic study [84].

Some recent developments in bacterial genomics, such as the so-called Genome-Organization Frameworks (GOFs), which are specific to each species and offer direction for sequence assembly and completion, have not been included in the existing tools. It is still necessary to include additional annotation data about non-coding RNAs, pseudogenes, and epigenetic elements in the appropriate software programs [85].

## 3 CHALLENGES IN PANGENOME

The issues surrounding gene assembly, annotation, categorization, and grouping can be effectively classified as the bioinformatics task of inferring a bacterial pangenome. As the dataset expands, minor errors within individual genomes will also accumulate. Methods are being developed to acknowledge and rectify these artifacts in response to the growing interest in bacterial pangenomes, making the subject more enticing [86].

### 3.1 Automatic Gene Annotation

One of the primary challenges in analyzing pangenomes is genome annotation, which can be improved in various ways. Computational tools and techniques for identifying genes often rely on a small portion of experimental evidence despite the vast increase in genome sequencing and assembly over the past decade. Annotation errors can arise from contamination, incorrect assembly, and the difficulties of automatically annotating draft genomes. These mistakes can potentially affect gene databases, especially when used to guide the annotation of new genomes. Predicting coding sequences (CDSs) in prokaryote gene annotation pipelines is typically done by a limited number of algorithms. However, these algorithms often struggle to account for fragmented assemblies, leading to incorrect and inconsistent annotations. Moreover, erroneous orthologs can occur due to out-of-frame mistakes. Well-known algorithms like Prodigal, Glimmer, and Gene Marks include a training phase to improve accuracy by considering the genome's annotation features. The pipeline also eliminates small open reading frames and flagged bogus CDSs [87].

### 3.2 Clustering Orthologs and Paralogs

Gene sequences need to be organized into orthologous and paralogous groups after annotation to better understand the common genes shared among genomes and the emergence of pangenomes. Paralogs arise from gene duplication events, while orthologs trace their origins back to speciation events. However, accurately generating these clusters can be challenging due to contamination, errors in gene annotation, and the vast variability in different gene families. To address this, techniques like blast and CD-HIT are commonly employed in pangenome clustering to detect homology and group genes based on pairwise distance matrices or initial clusters. However, a key distinction between these tools and pangenome clustering algorithms is their limited consideration of paralogous genes or the varying sequence identity within gene families [88]. This discrepancy in defining orthologs and paralogs can significantly impact pangenome size determination. To overcome these challenges, blast or similar alignment techniques are used to create distance matrices between gene pairs, accounting for sequence identity variations. Clustering approaches involve identifying triangles of greatest hits or utilizing the Markov clustering algorithm. Recent methods involve pre-clustering genes to eliminate redundancy before constructing distance matrices, considering the increasing size of datasets. Paralogs are typically identified using gene family phylogenies or gene synteny [89]. The Panaro algorithm utilizes gene synteny to detect broken genes, missing annotations, out-of-frame errors, and contamination. In order to ensure consistent annotations across genomes, the Peppen algorithm employs a clustering phase. While these techniques mitigate annotation errors, it is important to acknowledge that some false clusters may persist. Consequently, downstream analyses should consider these inaccuracies to avoid biasing our understanding of pangenome dynamics [90].

### 3.3 Intergenic Regions

Pangenome analysis tools primarily focus on protein-coding sequences, but this approach has limitations as it disregards non-coding RNAs and essential Intergenic Regions (IGRs) such as promoters, terminators, and binding sites for regulators. These features are known to be subject to selection and can have significant phenotypic effects. In contrast to protein identification methods, non-coding region annotation algorithms often rely on pre-existing feature models, thus avoiding genome-specific training issues. However, the presence of incorrect coding annotations and predicted non-coding RNAs can lead to similar challenges. Fragmented assemblies and contamination also contribute to the misannotation of non-coding sequences, representing substantial sources of errors [91].

## CONCLUSION

In conclusion, Many biologically coherent ensembles, such as taxonomic units or viral populations, have already had their DNA sequenced, which most certainly captures the majority of their frequently occurring genetic variation. Capturing 'all of genomes' in terms of genetic variety content and abundance is no longer a pipe dream; it will soon become a reality for many species, populations, and cancer genomes. Life sciences currently practice 'pan-genomics', which involves understanding all important genetic variations in a group of genomes of interest. In this article, we discuss organizing and analyzing vast amounts of knowledge, as well as dealing with potential implications in subsequent analyses. The present review is an overview of the available pan-genomic analysis tools.

In the future, sequencing prices will drop, and computational power will increase, promoting more pan-genomes analysis and aiding our understanding of the fundamental problem of the link between genes and species genesis.

## AUTHORS’ CONTRIBUTIONS

The authors confirm contribution to the paper as follows: study conception and design: P. Parvatikar; draft manuscript: A. Sarawad and S. Hosagoudar. All authors reviewed the results and approved the final version of the manuscript.

## Figures and Tables

**Table 1:** Examples of bacterial pangenome techniques.

**Organism**	**Approach**	**Genome**	**Core Size**
*Streptococcus agalactiae*	ORFsim, comb	8	1806
*Neisseria meningitidis*	ORFsim, comb	6	1337
*Borrelia burgdoferi*	ORFsim, comb	21	1200
*Escherichia coli*	ORFsim, comb	17	2344
*Enterococcus faecium*	ORFsim, comb	7	2172
*Yersinia pestis*	ORFsim, comb	14	3668
*Streptococcus pyogenes*	OG, Comb	11	1376
*Clostridium difficile*	OG, Comb	15	1033
*Lactobacillus paracasei*	OG	34	1800
*Campylobacter jejuni*	ORFsim, Ref	130	1042
*Campylobacter coli*	ORFsim, Ref	62	947
*Haemophilus influenzae*	FSM	13	1450

**Table 2:** Software tools for pan genomic studies.

**Name**	**Link**	**Platform**
**Panseq**	https://lfz.corefacility.ca/panseq/	Online Windows Linux
**PGAT**	http://nwrce.org/pgat	Online
**PanCGHweb**	http://bamics2.cmbi.ru.nl/websoftware/pancgh/pancgh_start.php	Online
**PGAP**	http://pgap.sourceforge.net/	Linux
**ITEP**	https://price.systemsbiology.net/itep	Linux
**CAMBer**	http://bioputer.mimuw.edu.pl/camber/index.html	Windows Linux
**Harvest**	https://github.com/marbl/harvest	Mac OSX Linux
**GET_HOMOLOGUES**	http://www.eead.csic.es/compbio/soft/gethoms.php	Mac OSX Linux
**PanCake**	https://bitbucket.org/CorinnaErnst/pancake/wiki/Home	Windows Linux
**PanGP**	http://PanGP.big.ac.cn	Windows Linux
**PANNOTATOR**	http://bnet.egr.vcu.edu/pannotator/index.html	Online
**Spine and AGEnt**	http://vfsmspineagent.fsm.northwestern.edu/index_age.html	Online Mac OSX Linux
**PanOCT**	https://www.jcvi.org/research/panoct	Unix/Linux
**panX**	https://pangenome.org/	-
**PanGP**	https://pangp.zhaopage.com/	Windows/Linux
**PanViz**	https://github.com/thomasp85/PanViz	Unix/Linux
**EUPAN**	https://cgm.sjtu.edu.cn/eupan/	Unix/Linux
**PanTools**	https://pantools.readthedocs.io/en/stable/	Unix/Linux, Mac OS, Windows
**Micropan**	https://cran.r-project.org/web/packages/micropan/index.html	Unix/Linux, Mac OS, Windows
**Pan-Tetris**	https://mybiosoftware.com/pan-tetris-inspection-of-gene-occurrences-in-pan-genome-table.html	On any machine with a Java VM installed
**Seq-seq-pan**	https://gitlab.com/rki_bioinformatics/seq-seq-pan	Unix/Linux
**NGSPanPipe**	https://github.com/Biomedinformatics/NGSPanPipe	-

## LIST OF ABBREVIATIONS

ANI: Average Nucleotide Identity
CDS: Coding Sequences
COGs: Clusters of Orthologous Groups
GOFs: Genome-Organization Frameworks
IGRs: Intergenic Regions
IWGSC: International Wheat Genome Sequencing Consortium
MDS: Multiple-Deletion Series
PATO: Pangenome Analysis Toolkit
SM: Snow Mold
SNPs: Single Nucleotide Polymorphisms
SVs: Significant Structural Variants
TIG3: Tazarotene-induced Gene 3
